# Acute development of a paradoxical pulse in a child

**DOI:** 10.1186/1749-8090-8-S1-O314

**Published:** 2013-09-11

**Authors:** R Leskovac, D Belina, D Anic, Z Kovac

**Affiliations:** 1 Department of Paediatric Cardiac Surgery, Zagreb University Hospital, Zagreb, Croatia; 2 Department of Pathophysiology, Zagreb University Hospital, Zagreb, Croatia

## Background

Paradoxical pulse is defined as a decrease of systolic blood pressure for more than 10 mmHg during inspiration. In this presentation we would like to show the abrupt development of paradoxical pulse observable by direct intra-arterial blood pressure measurement.

Acute development of a paradoxical pulse is important in recognizing tamponade development primarily due to acute bleeding. This can lead to acute cardiogenic shock and resuscitation.

A four month old infant underwentsurgery for Tetralogy of Fallot. Postoperative course was uneventful. On the second postoperative day mediastinal and pleural tubes were removed together with atrial and ventricular electrodes. Several seconds after removal, a normal and regular intra-arterial pressure wave on the monitor became highly distorted and irregular with abnormal pulse wave and the presence of tachycardia and hypotension. Bedside transthoracic echocardiogram (TTE) showed a 10 mm wide pericardial effusion.

Urgent resternotomy was done in the operating room andthe vast amount of coagulum with dark venous blood was found in the pericardium. Active bleeding of the right atrium auricula was verified at the site where a temporary unipolar electrostimulation electrode was previously positioned. The infant was extubated the same night and discharged from the hospital on the fourteenth day.

## Conclusion

Sudden presentation of 'unexpected irregularpulse' implied presence of acute pericardial tamponade that was a result of right atrial haemorrhage. Recognizing the paradoxical pulse in the ICU setting is important in order to avoidmorbidity in paediatric surgical patients.

